# Linking primary producer diversity and food quality effects on herbivores: A biochemical perspective

**DOI:** 10.1038/s41598-017-11183-3

**Published:** 2017-09-08

**Authors:** Vanessa Marzetz, Apostolos-Manuel Koussoroplis, Dominik Martin-Creuzburg, Maren Striebel, Alexander Wacker

**Affiliations:** 1 Institute of Biochemistry and Biology, Theoretical Aquatic Ecology and Ecophysiology, University of Potsdam, Am Neuen Palais 10, 14469 Potsdam, Germany; 2 Limnological Institute, University of Konstanz, Mainaustrasse 252, 78464 Konstanz, Germany; 3 Institute for Chemistry and Biology of the Marine Environment, University of Oldenburg, Schleusenstraße 1, 26382 Wilhelmshaven, Germany

## Abstract

Biodiversity can strongly influence trophic interactions. The nutritional quality of prey communities and how it is related to the prey diversity is suspected to be a major driver of biodiversity effects. As consumer growth can be co-limited by the supply of several biochemical components, biochemically diverse prey communities should promote consumer growth. Yet, there is no clear consensus on how prey specific diversity is linked to community biochemical diversity since previous studies have considered only single nutritional quality traits. Here, we demonstrate that phytoplankton biochemical traits (fatty acids and sterols) can to a large extent explain *Daphnia magna* growth and its apparent dependence on phytoplankton species diversity. We find strong correlative evidence between phytoplankton species diversity, biochemical diversity, and growth. The relationship between species diversity and growth was partially explained by the fact that in many communities *Daphnia* was co-limited by long chained polyunsaturated fatty acids and sterols, which was driven by different prey taxa. We suggest that biochemical diversity is a good proxy for the presence of high food quality taxa, and a careful consideration of the distribution of the different biochemical traits among species is necessary before concluding about causal links between species diversity and consumer performance.

## Introduction

Biodiversity is a major driver of ecosystem processes and has a wide array of effects on various ecosystem functions^1, 2^. For example, a high diversity of primary producers can stabilize primary production^1–4^ and is often accompanied by higher resource use efficiency and increased overall biomass production^4–7^. These effects at the producer level may also influence higher trophic levels through changes in nutritional quality^8, 9^. However, there is no clear consensus on how prey specific diversity is linked to nutritional quality. Previous studies have considered only single nutritional quality traits, such as phosphorus (P) or nitrogen (N) to carbon ratios^10, 11^, polyunsaturated fatty acid (PUFA)^12, 13^ or sterol concentrations^14, 15^. However, such consideration might be insufficient because several of these traits can simultaneously limit consumer performance^12, 16–18^. In general, the occurrence of such co-limitations could yield strong positive relationships between the biochemical diversity (a proxy of a balanced supply of co-limiting nutrients) of the prey community and consumer growth.

This consideration implies several scenarios of how prey diversity may correlate with nutritional quality and, ultimately, consumer growth. For example, co-limiting nutrients might be heterogeneously distributed among different phytoplankton species. Thus, in a more diverse phytoplankton community, the probability of co-occurring species with complementary nutritional traits increases, thereby promoting biochemical diversity and consumer growth (**scenario 1**, complementarity effect) and a positive correlation between consumer performance and food diversity would be observed. Another scenario could be that one or a few species provide all nutrients required for consumer growth and reproduction^19–21^. The presence of such superior nutritional quality species can alter the relationship between producer diversity and consumer growth. In a more diverse community the chance of having the high quality species would increase (**scenario 2**, sampling effect) and a positive correlation between consumer performance and food diversity would exist. Yet, at the same time, the relative contribution of the high quality species could be diluted in more diverse communities, depending on its competitive strength. The balance between these two counter-acting effects (higher chances to include high quality food vs. dilution of high quality food) could either yield null or negative correlations between consumer performance and food diversity (**scenario 3**).

To our knowledge, despite the large interest in understanding the effects of biodiversity on trophic interactions^9^, no study explicitly investigated the links between species diversity, biochemical diversity and consumer performance. Here we used the well- established model system consisting of the freshwater herbivore *Daphnia magna* and different phytoplankton species^9, 18, 22^. Based on the low quality species *Synechococcus elongatus* (non-toxic, well-ingestible, but lipid-deficient: lack of sterols and PUFA)^17^, included in all experimental setups, we established different phytoplankton communities differing in species diversity and thus evaluated the growth potential of *D. magna* on these phytoplankton communities focusing on the underlying mechanism between species and biochemical diversity. The phytoplankton species were selected as representatives of the major taxonomic groups commonly found in natural systems. The members within each taxonomic group share common biochemical traits, in particular their fatty acid and sterol composition^23–27^, and the chosen species can thus be considered as representative of the biochemical diversity found in nature. In order to account for the effects of phytoplankton species competition, e.g. for light or phosphorus, on the nutritional quality of the communities^28^ we allowed the species to develop their own communities rather than to artificially assemble mixtures of given species proportions to obtain a more realistic scenario.

## Materials and Methods

### Phytoplankton cultures

Eight freshwater phytoplankton species were used in this experiment. They were chosen based on taxonomic affiliation and specific traits such as distinct fatty acid and sterol profiles (Table 1). Each species was grown in monoculture and as several multi-species communities (Table 2). All monocultures and communities were cultivated simultaneously. Of all possible species combinations, 22 communities representing four species richness levels (2, 4, 6, 8) were chosen and used as the food source in the zooplankton growth experiment. In order to address complementarity of nutritional traits in natural communities and the improvement of food quality by diversity we inoculated the cyanobacterium *Synechococcus elongatus*, which is of low nutritional quality, (lack in PUFA and sterols, Table 1)^17^, in all communities. By always having a poor quality species present in the communities we ensured that animals are still able to respond to changes in food quality and species diversity at the upper end of their response curve. Species compositions were selected in a way that each species/group was present/absent in similar numbers of communities avoiding redundancy and creating a broad range of species and thereby biochemical traits (Table 1), which allowed for assessing taxonomic group or species - specific effects. All phytoplankton species were pre-cultured in P-limited (5 µM P_i_, in the form of K_2_HPO_4_) modified Woods Hole MBL medium^29^. To avoid a limitation by potassium (K), the final concentration of K was adjusted to 100 µM using potassium chloride (KCl). Aliquots of these pre-cultures were used to inoculate the experimental phytoplankton monocultures and communities. All experimental cultures started with a total biomass concentration of 1 mg C ∙ L^−1^; the communities were inoculated with equal carbon shares of each species. Carbon concentrations were estimated using previously determined carbon-extinction equations. All algal cultures were grown semi-continuously (0.15 d^−1^) in duplicates (except for community 4I, n = 4 replicates) at 20 °C and a 16/8 h light/dark cycle (light intensity: 256 µmol photons · m^−2^ · s^−1^) in 1 L Erlenmeyer flasks (see Supplementary Fig. S1) containing 600 mL of medium (5 µM P). From day 12 to 15 the removed fraction of the replicates of each community were combined daily and used as food for experimental *Daphnia* cultivation. On day 15, all phytoplankton cultures were harvested and prepared for chemical analysis, determination of cell numbers and biovolumes.

**Table 1:** Phytoplankton species used in this experiment with their abbreviations, taxonomic affiliation, origin, and main biochemical characteristics, such as fatty acids, the fatty acid diversity (H_FA_), and sterols (for additional information see Supplementary Fig. S2).

Name	Abbr.	Class	Origin	Fatty acids	H_FA_	Sterols
*Synechococcus elongatus* Nägeli	Syn	Cyanophyceae	SAG 89.79	no PUFA	1.30	no sterols
*Acutodesmus obliquus* (Turpin) Hegewald et Hanagata	Acu	Chlorophyceae	SAG 276-3a	rich in C18-PUFA	1.93	Chondrillasterol, Fungisterol
*Chlamydomonas reinhardtii* Dangeart	Chl	Chlorophyceae	SAG 11-32b	rich in C18-PUFA	1.74	Ergosterol, 7-dehydro-poriferasterol
*Cyclotella meneghiniana* Kützing	Cyc	Mediophyceae	SAG 1020-1a	rich in long- chain PUFA (> C18)	2.22	24-Methylene-cholesterol
*Stephanodiscus hantzschii* Grunow	Ste	Mediophyceae	University of Konstanz	rich in long- chain PUFA ( > C18)	1.99	24-Methylene-cholesterol
*Cryptomonas ovata* Ehrenberg	CryO	Cryptophyceae	SAG 979-3	rich in long- chain PUFA (EPA, DHA)	2.01	Stigmasterol, Epibrassicasterol
*Cryptomonas* sp. Ehrenberg	CryS	Cryptophyceae	26-80	rich in long- chain PUFA (EPA, DHA)	2.25	Stigmasterol, Epibrassicasterol
*Nannochloropsis limnetica* Krienitz, Hepperle, Stich, Weiler	Nan	Eustigmatophyceae	SAG 18.99	rich in long- chain PUFA (EPA, but no DHA)	2.02	Cholesterol, Isofucosterol, Sito-/clionasterol,

Note: *Acutodesmus obliquus* was previously named *Scenedesmus obliquus* (Turpin) Kützing. Abbreviations: polyunsaturated fatty acids (PUFA); eicosapentaenoic acid (EPA); docosahexaenoic acid (DHA).

**Table 2:** Species combinations for the 22 different phytoplankton communities (Com) containing 2, 4, 6 or 8 species.

Com		Syn	Acu	Chl	Cyc	Ste	CryO	CryS	Nan
2	A	x	x						
2	B	x		x					
2	C	x			x				
2	D	x				x			
2	E	x					x		
2	F	x						x	
2	G	x							x
4	A	x	x			x	x		
4	B	x	x			x		x	
4	C	x	x			x			x
4	D	x	x				x		x
4	E	x		x	x		x		
4	F	x		x	x			x	
4	G	x		x	x				x
4	H	x		x			x		x
4	I	x			x			x	x
4	J	x				x		x	x
6	A	x	x	x	x		x	x	
6	B	x	x		x	x	x	x	
6	C	x		x	x	x	x		x
6	D	x			x	x	x	x	x
8		x	x	x	x	x	x	x	x

Syn: *Synechococcus elongatus*, Acu: *Acutodesmus obliquus*, Chl: *Chlamydomonas reinhardtii*, Cyc: *Cyclotella meneghiniana*, Ste: *Stephanodiscus hantzschii*, CryO: *Cryptomonas ovata*, CryS: *Cryptomonas* sp., Nan: *Nannochloropsis limnetica*.

### Community composition

To determine the phytoplankton species composition, cell numbers and biovolumes in the communities, samples were fixed with Lugol’s iodine solution and counted using an inverted light microscope (Thalheim Spezial Optik, Pulsnitz, Germany and Axio Observer.A1, Carl Zeiss, Jena, Germany). The cyanobacterium was counted by epifluorescence microscopy (Axioskop 2, Carl Zeiss, Jena, Germany) after Acridin-orange staining (Merck, Darmstadt, Germany) under blue light (excitation: BP 450–490 nm; emission: BP 515–565 nm). Cell sizes of phytoplankton were measured and converted into biomass units according to Hillebrand *et al*.^30^. *Cryptomonas ovata* and *Cryptomonas* sp. were not clearly distinguishable via light microscopy and therefore consolidated as *Cryptomonas* spp.

### *Daphnia* culturing and determination of somatic growth rates

The zooplankton growth experiments were conducted with a clone of *Daphnia magna* (OER3–3, obtained from Dieter Ebert, Basel). A cohort of females was synchronized on a *Acutodesmus obliquus* diet in artificial *Daphnia* medium (ADaM)^31^ to release offspring at day 12 of phytoplankton growth. Shortly before the offspring were released, the females were transferred to ADaM containing *S. elongatus* (2 mg C ∙ L^−1^). Neonates born within 24 hours were isolated and randomly transferred to experimental vessels (7 individuals per vessel; n = 3) containing 200 mL food suspensions. The food suspensions were prepared by diluting the removed fractions of the respective phytoplankton communities, whose biomass ranged from 7.6 to 18.2 mg C ∙ L^−1^, with ADaM to a target concentration of 2 mg C ∙ L^−1^. Three subsamples, each consisting of 10 randomly selected individuals, were placed into pre-weighed aluminum boats, dried at 50 °C and weighed to determine the offspring dry mass at the beginning of the experiment. During the following 4 days of the experiment daphnids were transferred daily into fresh medium with freshly harvested food suspensions. After 4 days, the animals in each vessel were placed into pre-weighed aluminum boats, dried at 50 °C and weighed. The somatic growth rates were calculated using equation 1, where ${x}_{{t}_{0}}\,\,$is the dry mass at the beginning of the experiment, ${x}_{t}$ is the dry mass at the end of the experiment (µg), and ${\rm{\Delta }}t$ is the duration of the experiment in days.

1 $$g=\frac{\mathrm{ln}\,{x}_{t}-\,\mathrm{ln}\,{x}_{{t}_{0}}}{\,\Delta t}$$

### Chemical analyses

Each phytoplankton culture was analyzed for particulate organic carbon (C), particulate phosphorus (P), and fatty acids. Only monocultures and a community with the highest inoculated species richness could be analyzed for sterols because the available biomass limited the tests which could be performed (see below). For carbon determination, aliquots of the algal suspensions were filtered onto 25 mm precombusted glass fiber filters (GF ⁄F, Whatman, Dassel, Germany), dried at 50 °C and analyzed using an elemental analyzer (Euro EA 3000, HEKAtech GmbH, Wegberg, Germany). To measure particulate P concentrations, aliquots of the phytoplankton cultures were filtered onto polysulfone filters (25 mm, 0.45 µm; Pall Corporation, Port Washington, NY, USA). Phosphorus concentrations were determined using the molybdate blue reduction method^32^ after sulphuric acid treatment and oxidative hydrolysation with K_2_S_2_O_8_ at 121 °C and 104 kPa (autoclaving).

Samples for fatty acid determination were obtained by filtering 0.3 mg algal C onto 25 mm glass fiber filters (GF⁄F, Whatman). Filters were stored at −25 °C in 7 mL of dichloromethane-methanol (2:1 v⁄v) under a nitrogen atmosphere in glass tubes with Teflon seals. Lipids were extracted and transesterified into fatty acid methyl esters and then identified and quantified by gas chromatography (GC 6890 N, Network GC System, Agilent Technologies GmbH) according to Wacker *et al*.^33^. Sterols were extracted from GF/F filters loaded with aliquots of the algae cultures (1 mg C) and analyzed by gas chromatography (GC) and gas chromatography-mass spectrometry (GC-MS) as described in Martin-Creuzburg and Merkel^27^. Fatty acid and sterol concentrations were related to the respective carbon concentrations of the food suspensions.

### Calculations

The shares of the different species in the respective phytoplankton communities (based on biovolumes) were used to calculate the Shannon-Wiener diversity index^34, 35^, which takes into account how many species were present and how each species was represented in the community.

The diversity of the fatty acid composition within a community was also calculated according to the Shannon-Wiener index. This calculation included the proportions of 17 fatty acids that were most common in phytoplankton species used here (see Supplementary Fig. S2).

Total sterol concentrations in communities were calculated using sterol concentrations from algae monocultures, because the harvested biomass was not sufficient to determine the sterol concentration of every community. The carbon-based sterol concentrations of the monocultures were converted into biovolume-based sterol concentrations (*M*_*j*_) and related to the microscopically determined biovolumes (*BV*_*j*_) of each species (*j*) and the number of species in the community (I) to calculate the total expected sterol concentration (*E*) of the whole community (Equation 2)^28^.

2 $$\,E=\,\sum _{i=1}^{I}(B{V}_{j}\,\cdot {M}_{j})$$

The calculated sterol concentration was then converted back to carbon units. To verify the validity of this procedure, we analyzed the total sterol concentration of a community inoculated with the highest species richness and compared these values with total sterol concentrations calculated for this particular community. Both procedures revealed comparable total sterol concentrations (calculated: 4.68 ± 1.07; measured: 4.43 ± 0.27 µg mg ∙ C^−1^), indicating that the total sterol concentrations of the different communities were reliably estimated.

### Data analyses

The relationship between *Daphnia* growth rates and the species diversity as well as fatty acid diversity indices were analyzed using Pearson correlations. To investigate the determinative variables for the growth rate response of *D. magna*, a principal component analysis (PCA) was conducted. Scaled values of the Shannon-Wiener index of phytoplankton diversity were included in the PCA. Additionally, we included the P:C ratio, estimated total sterol concentrations, and concentrations of several prominent fatty acids as indicators of nutritional quality. The following fatty acids and fatty acid sum parameters were considered: the sum of saturated fatty acids (SFA), the unsaturated fatty acid oleic acid (OLA, 18:1n-9), the polyunsaturated fatty acids linoleic acid (LNA, 18:2n-6), α-linolenic acid (ALA, 18:3n-3), arachidonic acid (ARA, 20:4n-6), and eicosapentaenoic acid (EPA, 20:5n-3). Data scores of PC were additionally analyzed for their relationship to *Daphnia* growth rates using analysis of co-variance (ANCOVA). All calculations and statistical analyses were carried out using the statistical software package R version 3.3.2 (R Development Core Team 2010, R Foundation for Statistical Computing, Vienna, Austria).

### Data availability

The datasets generated and analyzed during the current study are available from the corresponding author on reasonable request.

## Results

**Figure 1:**
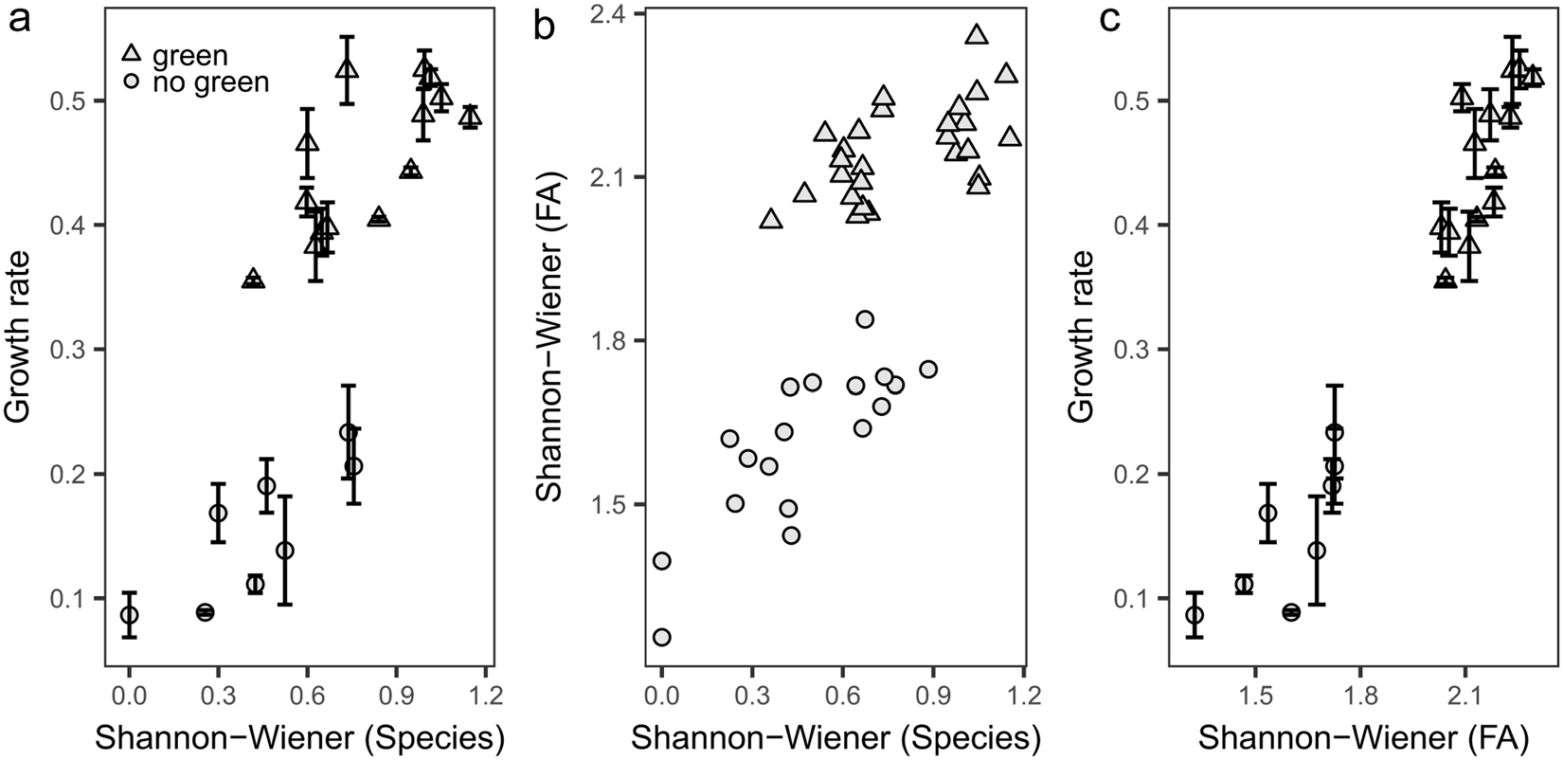
Correlation of (**a**) the Shannon-Wiener index of phytoplankton species diversity with *Daphnia* growth rates (R^2^ = 0.6), shown as mean ± SD (**b**) the Shannon-Wiener index of species and most common fatty acids (R^2^ = 0.62) and (**c)** Shannon-Wiener index of fatty acids (FA) with *Daphnia* growth rates (R^2^ = 0.9). The different symbols indicate communities with and without Chlorophyceae (triangles and dots, respectively). *Daphnia* growth rates (n = 3 replicates) were correlated with the average of the Shannon-Wiener indices, as the replicates of the communities were pooled as food source. For correlation between both Shannon-Wiener indices individual values of the cultures were used.

**Figure 2:**
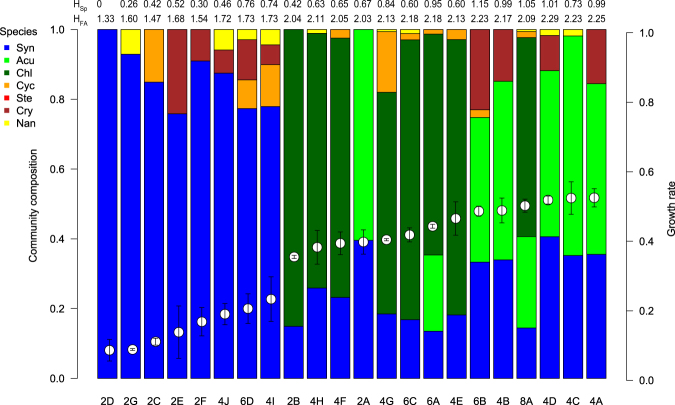
Relative phytoplankton community composition and *Daphnia* growth rate. Mean relative phytoplankton composition of communities which were used as food in the *Daphnia* growth experiments. Numbers below bars indicate the number of inoculated species and, combined with the letters, relate to the species combinations given in Table 2 at day 15 of the experiment. Phytoplankton communities were sorted according to their food quality for *Daphnia*; somatic growth rates (second y-axis and white circles; mean ± SD, n = 3) increasing from left to right. Numbers above bars indicate the Shannon-Wiener Index for species (H_Sp_) and fatty acid (H_FA_) diversity. Species abbreviations are *Synechococcus elongatus* (Syn), *Acutodesmus obliquus* (Acu), *Chlamydomonas reinhardtii* (Chl), *Cyclotella meneghiniana* (Cyc), *Stephanodiscus hantzschii* (Ste), *Cryptomonas ovata* and *Cryptomonas* sp. (consolidated as Cry), *Nannochloropsis limnetica* (Nan).

Higher phytoplankton species diversity resulted in higher zooplankton growth rates (R^2^ = 0.6; Fig. 1a) and higher fatty acid diversity in the phytoplankton communities (R^2^ = 0.62; Fig. 1b). The Shannon-Wiener index of dietary fatty acids, i.e. fatty acid diversity, was strongly correlated (R^2^ = 0.9) with *Daphnia* growth rates (Fig. 1c). The remaining variance of *Daphnia* growth rates can be explained by species identity and other food quality traits (such as sterols, C18 and C20 PUFA) of the species in the communities (Fig. 2, Table 1). Overall, phytoplankton communities in which green algae were competing against the cyanobacterium *S. elongatus* allowed for high *Daphnia* growth rates (values ranged from 0.36 ± 0.01 d^−1^ to 0.53 ± 0.03 d^−1^). In the absence of green algae, the communities were dominated by the sterol- and PUFA-free cyanobacterium *S. elongatus* and, consequently, *Daphnia* growth rates were low (values ranged from 0.09 ± 0.03 d^−1^ to 0.23 ± 0.06 d^−1^; Fig. 2). Phytoplankton species rich in long-chain PUFA, such as *Cryptomonas* spp., *Cyclotella meneghiniana*, and *Nannochloropsis limnetica*, contributed at most 30% but usually less than 20% to the species composition in most communities (Fig. 2). *Stephanodiscus hantzschii* was outcompeted and not found in any community.

### PCA

**Figure 3:**
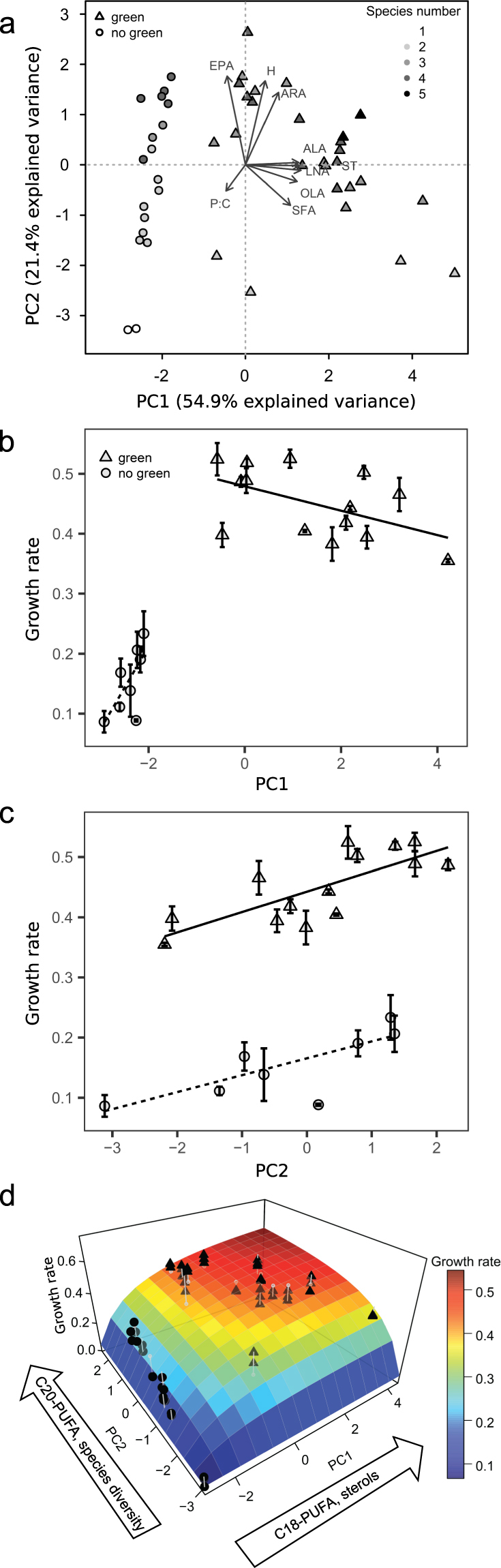
(**a**) Principal component analysis including Shannon-Wiener index of phytoplankton species diversity (H), molar P:C ratio, total sterols (ST), saturated fatty acids (SFA), oleic acid (OLA), α-linolenic acid (ALA), linoleic acid (LNA), arachidonic acid (ARA), and eicosapentaenoic acid (EPA) as variables. Symbols used as in Fig. 1. Grey scale indicates the number of species present in the phytoplankton communities at day 15. *Daphnia* growth rate versus PC1 (**b**) and PC2 (**c**). Lines show separate regression lines for communities with (top bold line) or without green algae (lower dotted line). With PC1 the variables ST, LNA, ALA, OLA and SFA; and with PC2 H, ARA, and EPA increase. The molar P:C quota slightly decreases with both, PC1 and PC2. The three dimensional growth response surface including both PC axes (**d**) shows our conceptual idea how the complementary distribution of nutritional traits in the phytoplankton communities may have driven the co-limitation of consumer growth. Growth rate increased with species diversity in communities without chlorophytes (dots) because of a higher probability to include C20 PUFA-rich species. Incorporating chlorophytes in communities (triangles) increased the supply of co-limiting nutrients, and the fatty acid diversity increased further as the proportion of C20 and C18 PUFA became more balanced. The sterol-rich chlorophytes allowed for saturation of consumer demand for sterols, thereby increasing consumer growth further and shifting it to single C20 PUFA limitation.

The relationship of *Daphnia* growth rates to single fatty acids or sterols was poor (Supplementary Figure S3). Hence, the relationship between *Daphnia* growth rates and the availability of essential nutrients in the phytoplankton communities was further analyzed using principal component analysis (PCA, Fig. 3). The first two principal components (PC) of the PCA already explained > 75% of total variance of the data of the phytoplankton communities (see Supplementary Table S1). Most of the variability was explained by PC1 (54.9%), which positively correlated with the estimated total sterol concentrations, ALA, LNA, oleic acid, and SFA. PC1 also separated communities based on the presence or absence of green algae (right vs. left quadrants of Fig. 3a). PC2 explained 21.4% of variance of data and correlated positively with the Shannon-Wiener index and the concentrations of EPA and ARA (see Supplementary Table S1). The Shannon-Wiener diversity index (H) is strongly influenced by the number of species and the PC2 thus separated the communities with a higher number of species in the upper quadrants from a lower number of species in the lower quadrants (Fig. 3a). The molar P:C ratio contributed only marginally to PC1 or PC2 (see Supplementary Table S1). Molar P:C ratios of the phytoplankton communities ranged from 0.0028 to 0.0053 and thus were mostly above the proposed thresholds for P-limited growth of *Daphnia* (~0.003)^11^.

Correlating *Daphnia* growth rates with data scores of the principal components (Fig. 3b,c) revealed that two groups of *Daphnia* growth rates were delineated by PC1, which represents sterols and ALA. Depending on the absence or presence of the green algae, *Daphnia* growth rates significantly increased with PC1 or stayed constant (Fig. 3b; ANCOVA: F_1,58_ = 187.62, p < 0.001; interaction: F_1,58_ = 10.82, p < 0.01). *Daphnia* growth rates generally increased also with PC2, representing a measure of the Shannon-Wiener index and the concentrations of dietary EPA and ARA (ANCOVA: F_1,58_ = 162.42, p < 0.001). The presence of green algae (F_1,58_ = 414.2, p < 0.001) led to a parallel shift with higher *Daphnia* growth rates but no change in the slope (interaction: F_1,58_ = 0.38, p = 0.54, Fig. 3c).

## Discussion

We demonstrate here that phytoplankton biochemical traits and their distribution among species can, to a large extent, explain consumer growth and its apparent dependence on phytoplankton species diversity. First evidence for a functional link between zooplankton nutrition and phytoplankton diversity was provided in a similar experimental setup by Urabe and Waki^8^. Subsequent studies confirmed that zooplankton growth is linked to phytoplankton species richness^9^. However, none of these studies experimentally considered nutritional quality traits other than C:P ratios. Although a role of essential biochemical nutrients was suspected already^8, 9^ there was no attempt to explicitly link biochemical nutrient diversity to consumer growth. In our experiment, phytoplankton cultures were inoculated with various species belonging to major taxonomic groups that are commonly found in nature. By letting the species grow and compete with each other we generated simplified semi-natural communities in which nutritional traits such as sterols, C18 PUFA and C20 PUFA, were complementary distributed among the main groups of phytoplankton species.

The communities with which *Daphnia* achieved high growth rates were generally the communities with high fatty acid diversity (Fig. 2). This result suggests that consumer growth is promoted by a more balanced dietary fatty acid supply. Furthermore, the highest growth rates and the highest fatty acid diversity were achieved when the phytoplankton communities included green algae (Fig. 1c). The PCA revealed that communities containing green algae were associated with high proportions of sterols and C18 PUFA (Fig. 3, PC1). The second PC (PC2) was associated with high concentrations of C20 PUFA (EPA, ARA, Fig. 3), mainly contributed by *C. meneghiniana*, *Cryptomonas* spp. and *N. limnetica. Daphnia* growth rates increased with the phytoplankton community PC2 scores, suggesting that the animals were limited by dietary C20 PUFA when feeding on at least some of these communities. It is well established that PUFA and sterols can simultaneously limit the growth of *Daphnia* in the laboratory^17, 36^ and the field^37^. The correlations between consumer growth and PC1 and 2 scores describing a co-limitation by sterols/C18 PUFA (associated with PC1, Fig. 3b) and C20 PUFA (associated with PC2, Fig. 3c) suggest that the nutritional traits of the resultant phytoplankton communities were co-limiting *Daphnia* growth (Fig. 3d).

In communities without chlorophytes, the beneficial effect of species diversity on consumer growth can be explained by the higher probability of ingesting C20 PUFA-rich species. By contributing additional fatty acids, these species also increase the fatty acid richness, thereby explaining the positive correlation between species and fatty acid diversity indices. In these communities, C20 PUFA-rich species supply all the known potentially co-limiting nutrients (i.e. sterols and C20 PUFA), since the basal food *S. elongatus* contains none of them. Yet, our data suggest that the supply with these co-limiting nutrients was not sufficient for saturating consumer growth. By incorporating chlorophytes in the communities, the fatty acid diversity increased further as the proportion of C20 and C18 PUFA became more balanced (fatty acid evenness increased). More importantly, however, chlorophytes, which are rich in sterols, presumably saturated the consumer sterol demands, thereby increasing consumer growth further and shifting the limiting nutrient to a mere C20 PUFA limitation.

Studies which have investigated the foraging behavior of generalist herbivores suggested that mixed complementary diets are preferably consumed, as they provide all necessary nutrients, may dilute toxic substances, and may even improve digestion^38–41^. Overall, there is strong evidence that mixed diets enhance the performance of terrestrial and aquatic herbivores^38, 42–44^, thus emphasizing the significance of primary producer diversity for higher trophic levels. Yet in most cases the performance on mixed diets is not better than that on the single most optimal species^20, 21^. Although we have not considered growth on single species here, our results also indicate that the apparent diversity effects on consumer growth are to a large extent driven by few biochemically superior species i.e. *C. meneghiniana*, *Cryptomonas* spp. and *N. limnetica*. In natural systems, a less diverse community dominated by PUFA- and sterol-rich diatoms and cryptophytes may already be of excellent nutritional value for consumers^37^. Possibly, in such communities the biochemical diversity is higher since these species increase both the richness and the evenness of the fatty acid pool. Furthermore, overall species diversity based on indices or numbers is likely to change seasonally and annually and depending on species-specific nutritional traits, the availability of essential lipids may change. Even, if the species diversity index does not change, the fatty acid profiles might be significantly altered. While species replacing each other during the season might lead to a relatively constant general species diversity index, the replacement of species with particular species-specific fatty acid composition will generate completely different fatty acid pattern in the communities^45^.

However, we also found some evidence for resource complementarity. Nutritionally superior species contribute much more C20 PUFA than sterols and when diluted in a community dominated by *S. elongatus* they cannot fully saturate all co-limiting nutrient demands of the consumers. In such cases, chlorophytes, which are rich in sterols, can act as complementary food species.

In conclusion, our results provide a possible explanation for the functional link between zooplankton nutrition and phytoplankton diversity found in previous studies^8, 9^. However, prey biochemical traits might not be the only drivers of consumer performance. From a more general perspective, the phytoplankton species may also differ in defense traits such as size and cell morphology, directly influencing trophic interactions^46–48^. Although the distribution of these traits among species might drive diversity effects in ways we have not considered here, our study clearly underlines the important role of nutritional traits for the understanding of diversity effects.

## Electronic supplementary material

Supplementary information
