# Supplementary material for: Linking primary producer diversity and food quality effects on herbivores: A biochemical perspective
Source: Sci Rep. 2017 Sep 8;7:11035. doi: 10.1038/s41598-017-11183-3 (PMC5591185; doi:10.1038/s41598-017-11183-3)
Supplement: Supplementary file 1 — Supplementary information [file 41598_2017_11183_MOESM1_ESM.docx]

**Linking primary producer diversity and food quality effects on herbivores: A biochemical perspective**

Vanessa Marzetz^*1^, Apostolos-Manuel Koussoroplis^1^, Dominik Martin-Creuzburg^2^, Maren Striebel^3^, and Alexander Wacker^1^

^1^ Institute of Biochemistry and Biology, Theoretical Aquatic Ecology and Ecophysiology, University of Potsdam, Am Neuen Palais 10, 14469 Potsdam, Germany

^2^ Limnological Institute, University of Konstanz, Mainaustrasse 252, 78464 Konstanz, Germany

^3^ Institute for Chemistry and Biology of the Marine Environment, University of Oldenburg, Schleusenstraße 1, 26382 Wilhelmshaven, Germany

The following information is in support of an article published in Scientific Reports

**Supplementary**


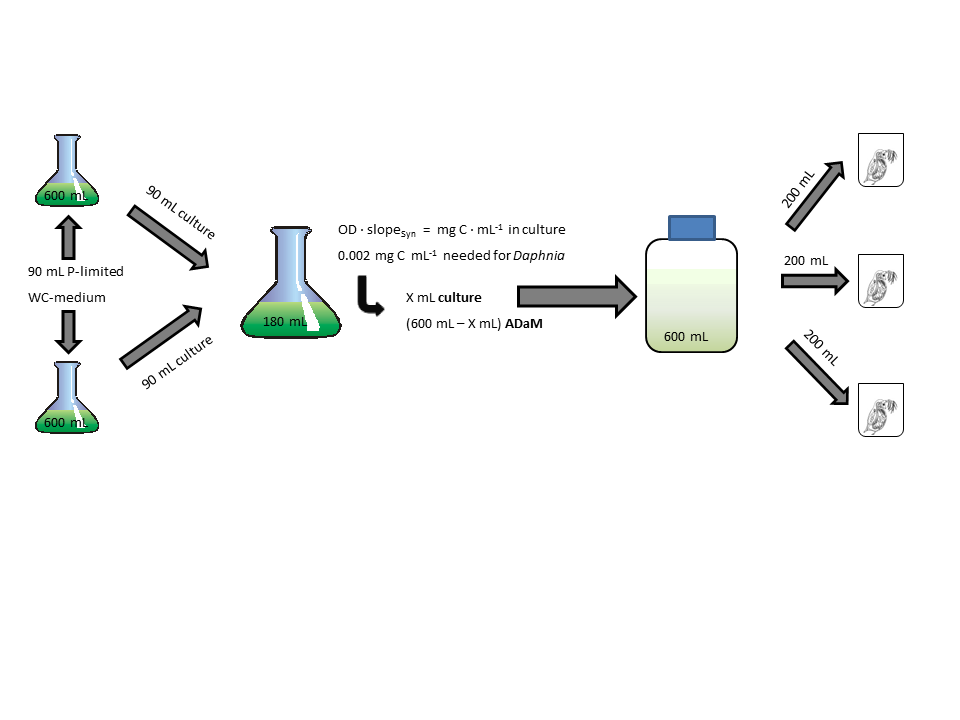


Fig. S1: Experimental overview showing the daily routine from left to right. During the whole experiment the phytoplankton communities were diluted daily. From day 12 on the exchanged volume of both replicate cultures were pooled and used for preparation of the food suspension. This was diluted with ADaM to a concentration of 2 mg C ∙ L^-1^ in 600 mL, equally divided to three vessels and seven *Daphnia magna* neonates were added. Those daphnids were then transferred daily into a new food suspension.


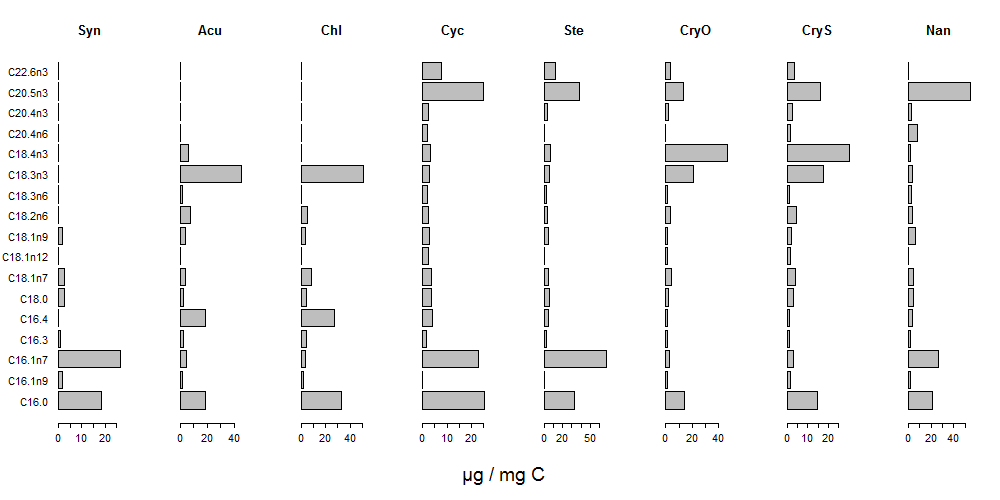


Fig. S2: Fatty acid concentrations (µg ∙ mg C^-1^) of the phytoplankton species used in this experiment. Syn: *Synechococcus. elongatus*, Acu: *Acutodesmus obliquus*, Chl: *Chlamydomonas reinhardtii*, Cyc: *Cyclotella meneghiniana*, Ste: *Stephanodiscus hantzschii*, CryO: *Cryptomonas ovata*, CryS: *Cryptomonas* sp., Nan: *Nannochloropsis limnetica.*


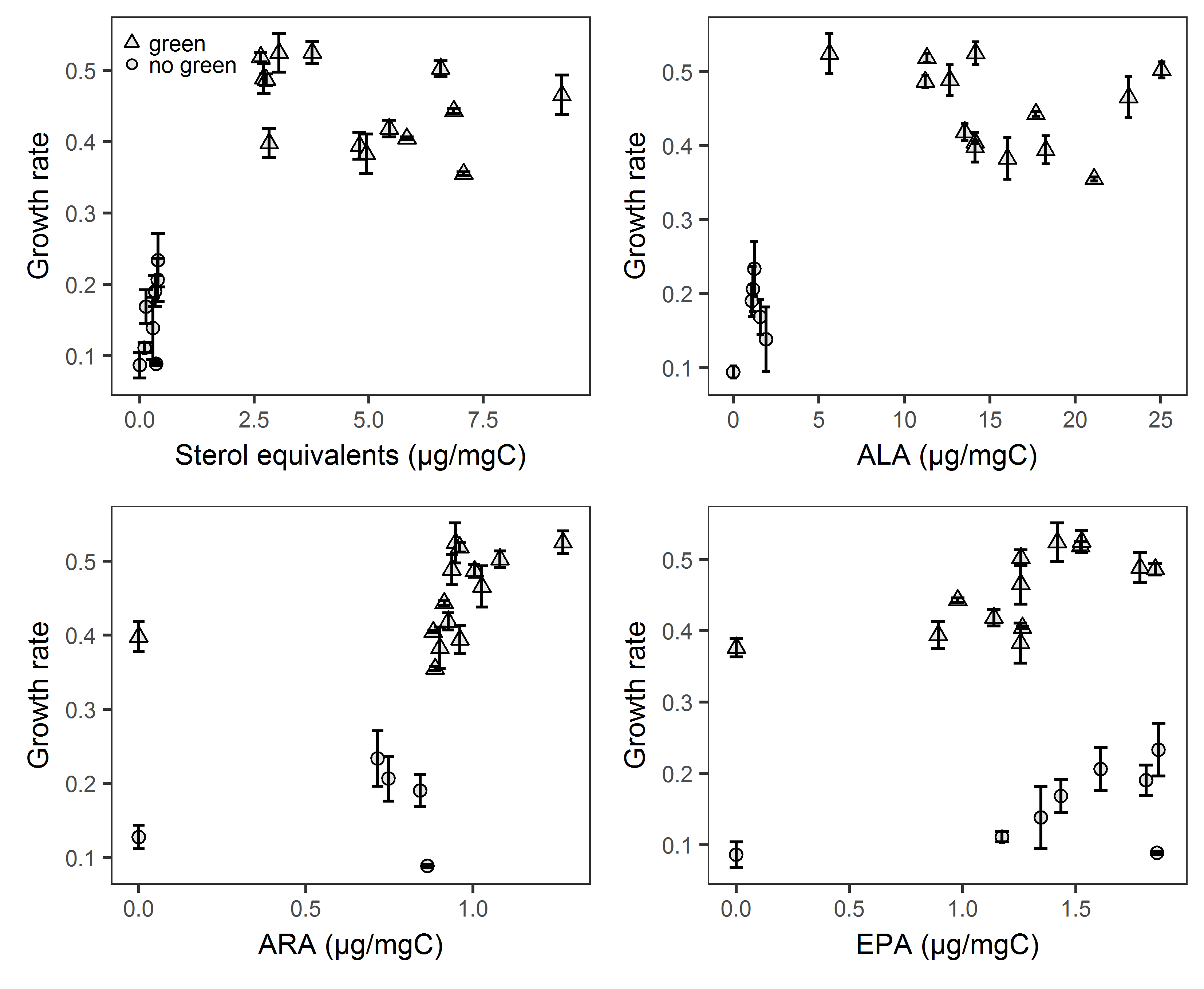


**c d**

**a b**

Fig. S3: *Daphnia* growth correlated to the concentrations of (a) sterol equivalents, (b) alpha-linolenic acid (ALA), (c) arachidonic acid (ARA), and (d) eicosapentaenoic acid (EPA). Symbols indicate if green algae were present (triangles, green) or absent (circles, no green).

Table S1: Summary of principal component analysis (PCA) using P:C ratio, concentrations of fatty acids, total sterols and the Shannon-Wiener index as variables.

| PCA parameters | PC1 | PC2 | PC3 |
| --- | --- | --- | --- |
| Eigenvalue | 4.94 | 1.93 | 0.95 |
| Variance explained (%) | 54.87 | 21.44 | 10.59 |
| Component loadings |  |  |  |
| H | 0.16 | 0.56 | 0.20 |
| P:C (mol:mol) | -0.16 | -0.17 | 0.92 |
| SFA | 0.36 | -0.27 | 0.17 |
| Oleic acid | 0.41 | -0.11 | -0.16 |
| LNA | 0.44 | -0.04 | -0.03 |
| ALA | 0.43 | 0.01 | 0.17 |
| ARA | 0.26 | 0.48 | 0.02 |
| EPA | -0.15 | 0.59 | 0.11 |
| ST | 0.43 | -0.01 | 0.15 |
|  |  |  |  |

Note: Abbreviations are of H: Shannon-Wiener index; SFA: saturated fatty acids; LNA: linoleic acid; ALA: α-linolenic acid; ARA: arachidonic acid; EPA: eicosapentaenoic acid; and ST: Total sterols.
